# Efficacy of empagliflozin as adjunctive therapy to citalopram in major depressive disorder: a randomized double-blind, placebo-controlled clinical trial

**DOI:** 10.1186/s12888-024-05627-0

**Published:** 2024-02-26

**Authors:** Atefeh Zandifar, Maryam Panahi, Rahim Badrfam, Mostafa Qorbani

**Affiliations:** 1 Social Determinants of Health Research Center, Alborz University of Medical Sciences, Karaj, Iran; 2 Department of Psychiatry, Imam Hossein Hospital, Alborz University of Medical Sciences, Karaj, Alborz Iran; 3 Faculty of Medicine, Alborz University of Medical Sciences, Karaj, Iran; 4 Non-communicable Diseases Research Center, Alborz University of Medical Sciences, Karaj, Iran; 5 Chronic Diseases Research Center, Endocrinology and Metabolism Research Institute, Tehran University of Medical Sciences, Tehran, Iran

**Keywords:** Empagliflozin, Citalopram, Major depressive disorder, Neuromodulating, Anti-inflammatory

## Abstract

**Background:**

Major depressive disorder is one of the most common psychiatric disorders, which is associated with a high disease burden. Current treatments using antidepressants have limitations, so using medication with neuromodulating and anti-inflammatory properties alongside them could be helpful. In a clinical trial, we studied the effectiveness of empagliflozin, a blood sugar-lowering drug, as an adjunctive therapy to reduce the severity of depression symptoms.

**Methods:**

A number of outpatients with moderate to severe depression (Hamilton Depression Rating Scale (HDRS) > = 17) who were not under related medication or had not taken medication for at least the last two months, had an age range of 18–60 years and had written informed consent to enter the study (*N* = 90) were randomly divided into two groups receiving placebo or empagliflozin (10 mg daily) combined with citalopram (40 mg daily) based on permuted block randomization method in an 8-week randomized, double-blind, placebo-controlled clinical trial. They were evaluated using the HDRS in weeks 0, 4, and 8.

**Results:**

HDRS scores were equal to 28.42(± 3.83), 20.20(± 3.82), and 13.42(± 3.42) in the placebo group during weeks 0,4, and 8, respectively. These scores were 27.36(± 3.77), 13.76(± 1.40), and 7.00(± 1.13), respectively, for the group treated with empagliflozin. Compared to the control group, patients treated with empagliflozin using repeated-measures ANOVA showed greater improvement in reducing the severity of depression symptoms over time (*p* value = 0.0001).

**Conclusions:**

Considering the promising findings in this clinical trial, further study of empagliflozin as adjunctive therapy in MDD with larger sample sizes and longer follow-ups is recommended.

## Introduction

Major Depressive Disorder (MDD) is a severe mental illness that can be debilitating for individuals [1, 2]. It is considered one of the most common psychiatric disorders, leading to high incidence, severity, and recurrence rates [3]. MDD affects an estimated 300 million people worldwide every year, accounting for the highest number of years of disability among psychiatric disorders [4]. This means that 5–6% of the world’s population is affected by MDD each year, while 11–15% of people experience it at some point in their lifetime [5].

Selective serotonin reuptake inhibitors (SSRIs) are a type of antidepressant that are commonly prescribed as the first-line of treatment for major depressive disorder (MDD). The most commonly prescribed SSRIs include paroxetine, fluoxetine, sertraline, and citalopram [6]. These medications work by blocking the 5-hydroxytryptamine (5-HT) transporter in presynaptic neurons, which helps to increase the levels of serotonin in the brain [7]. However, it’s important to note that SSRIs are not always effective for everyone. Studies have shown that only about 60% of MDD cases respond to treatment with SSRIs [8]. Additionally, high doses of SSRIs are not recommended as a routine treatment due to the risk of side effects [9]. It’s also worth noting that the effectiveness of SSRIs usually occurs in the lower range of their licensed dose, and increasing the dose does not significantly increase this effectiveness [10].

In recent years, there has been a focus on modern neurobiological approaches to better understand the pathophysiology and effective treatment of depression. This is due to gaps in knowledge in this field [11]. Induction of oxidative pathways, which is associated with immune inflammatory response, may play an important role in these pathogenic mechanisms [12]. Additionally, there has been an observed increase in the level of inflammatory cytokines and the induction of their signaling pathways in the brain and peripheral blood in important groups of patients with depression [13].

Based on recent evidence, insulin resistance plays a crucial role in depression’s clinical manifestations, pathophysiology, and treatment response [14]. Insulin levels and insulin resistance-related indices increase during acute episodes of depression, according to laboratory studies [15].

The use of add-on treatments to the standard therapy of MDD, with such an approach, has been the attention of experts in recent years [16, 17]. Metformin was found to have anti-inflammatory, antioxidant, and neuroprotective properties. In a 12-week clinical trial, 80 outpatients with MDD received fluoxetine (20 mg daily) and either metformin (1000 mg daily) or a placebo. The group receiving metformin had significantly higher response and remission rates, along with decreased serum levels of inflammatory factors and increased levels of biological markers such as brain-derived neurotrophic factor (BDNF) and serotonin [18].

In a clinical trial, the use of pioglitazone, an antidiabetic drug that acts as a synthetic agonist of peroxisome proliferator-activated nuclear receptor (PPAR)-gamma, was found to have properties such as anti-inflammatory, neuroprotective and anti-excitotoxic effects. It was used as an adjunctive treatment to citalopram in patients suffering from moderate-to-severe Major Depressive Disorder (MDD) and without underlying diabetes. This treatment showed significant improvement in depressive symptoms as compared to placebo. In this clinical trial, a group of 20 patients with an HDRS score of ⩾22, who were already under treatment with 20 mg citalopram daily, were given 15 mg pioglitazone twice a day for a period of 6 weeks. The results showed that the patients who received pioglitazone treatment had a significant reduction in their HDRS score over time, with a higher percentage of early improvement, response (at week 6), and remission compared to those who received a placebo [19].

Empagliflozin is a potent inhibitor of sodium-glucose co-transporter 2 (SGLT2) that selectively targets this protein [20, 21]. This results in an increase in the excretion of glucose through urine [22]. Empagliflozin is a suitable treatment option for patients with type 2 diabetes mellitus due to its high-capacity transport of glucose protein and low affinity to plasma proteins [23]. Additionally, the use of Empagliflozin has been associated with various benefits in heart function and cardiovascular problems such as heart failure, regardless of glucose tolerance status [24, 25]. Empagliflozin has been found to alleviate cerebral ischemia/reperfusion damage in animal models by activating the HIF-1α/VEGF signaling pathway and suppressing the oxidative-inflammatory-apoptosis pathway [26, 27]. Some animal studies have shown that activating the HIF-1α/VEGF signaling pathway can also improve depression symptoms [28, 29]. This suggests that influencing this pathway could be a potential goal for developing new treatments for depression.

It has been discovered that SGLT2 inhibitors group of drugs can reduce serum ACTH levels. This is due to the common mechanism of action of this group of drugs [30] On the other hand, patients with MDD have a dysfunction in the hypothalamic-pituitary-adrenal (HPA) axis which results in an increase in the level of this hormone [31, 32] Therefore, it is assumed that this group of drugs may have a positive effect on improving depressive symptoms [33].

Empagliflozin has been shown to reduce pro-inflammatory cytokines in animal studies, which may lead to a secondary effect of reducing neuroinflammation and neurodegeneration. This is significant as there is evidence suggesting the role of inflammatory factors in the exacerbation of MDD [34]. Therefore, the use of this medication may have potential benefits in controlling the underlying pathophysiology of MDD [35].

This particular feature of empagliflozin is linked to its ability to increase the expression of PPAR-γ, nuclear factor erythroid 2-related factor 2 (nrf2), and their target gene Hmox-1, which helps combat oxidative stress and inflammation [36]. This can be a contributing factor to the benefits of empagliflozin in improving cerebrovascular flow, and may also be a promising avenue for other benefits related to the improvement of depressive symptoms in patients with MDD [37] Moreover, other animal studies have highlighted the role of Nrf2-mediated antioxidants in the prevention of depression [38, 39].

ŞAHİN et al. conducted a study to examine the effects of SGLT2 inhibitors on mental health. They evaluated the impact of empagliflozin (10 mg daily) or dapagliflozin (10 mg daily) on the quality of life, anxiety, and sleep quality of a group of patients with type 2 diabetes mellitus. The clinical trial lasted for 12 weeks, and at the end of it, an improvement in the quality of life was reported among patients who received empagliflozin/dapagliflozin compared to the control group [40].

To the best of our knowledge, there haven’t been any clinical studies conducted to investigate the effectiveness of empagliflozin as an additional therapy to address depressive symptoms in patients. However, the underlying mechanisms associated with the action of empagliflozin raise the hypothesis that it may help improve depressive symptoms in individuals diagnosed with MDD. In this 8-week clinical trial, we aimed to evaluate the effectiveness of empagliflozin as an adjunctive therapy for patients with moderate to severe depression who were already undergoing treatment with citalopram. The trial involved a placebo-controlled approach to ensure accurate results.

## Materials and methods

This study is an 8-week randomized, double-blind, placebo-controlled, parallel-group clinical trial that evaluates the effect of adjunctive treatment with empagliflozin compared to placebo (combined with citalopram) in the treatment of patients with major depressive disorder. This study was conducted from February 2022 to August 2022.

### Participants

The participants in the study were selected from outpatients referred to the psychiatric clinic of Imam Ali Hospital, Alborz, Iran. The diagnosis of MDD based on the Diagnostic and Statistical Manual of Mental Disorders-Fifth Edition (DSM-5) criteria was made for them by two experienced psychiatrists who are faculty members of the department of psychiatry of Alborz University of Medical Sciences, following an independent structured interview.

The inclusion criteria included all the above-mentioned patients with moderate to severe depression (Hamilton Depression Rating Scale (HDRS) > = 17) who had an age range of 18–60 years and had written informed consent to enter the study.

Exclusion criteria included the comorbidity of other major psychiatric disorders (schizophrenia, bipolar disorder, anxiety disorders, obsessive-compulsive disorder) and psychotic symptoms in patients. Any substance use in the last 3 months was another exclusion criterion of the study (except nicotine and caffeine use in the absence of any dependence). Unwillingness to provide written consent to participate in the study, not being available during the trial, having any concurrent neurological disorder, having diabetes (type 1 or 2 treated with any blood sugar lowering drug), active liver and kidney disease, history of any chronic disease (kidney, liver, cardiopulmonary, etc.), history of malignancy, immune deficiency, major surgery, autoimmune disorder, uncontrolled hypothyroid or hyperthyroid situation, any cardiovascular disease or interventions, history of chronic or uncontrolled blood pressure, hypotension, active or recent genitourinary infection (within the last 3 months), pregnancy or breastfeeding, history (during the last month) of receiving antidepressants and other psychotropic medication or recent electroconvulsive therapy (ECT) during the last 2 months, history of receiving pioglitazone and other SGLT-2 inhibitor medications such as canagliflozin and dapagliflozin during the last 3 months, history of receiving Angiotensin-Converting Enzyme (ACE) inhibitors, Hydralazine, long-acting nitrates, beta blockers or Angiotensin Receptor Blockers (ARBs), Rasagiline, amlodipine, furosemide and linezolid during the last 3 months, history of allergy to empagliflozin and any other drug of this category were other exclusion criteria.

Having diabetes or pre-diabetic conditions managed by an internist-endocrinologist without concurrent treatment with hypoglycemic drugs was not a criterion for exclusion from the study. Considering that the glucose-lowering effect of empagliflozin is low in normoglycemia, the risk of hypoglycemia with these agents is very low [41].

### Measures

#### Hamilton Depression Rating Scale (HDRS)

HDRS is the most widely used scale for selecting and following-up patients with depression in clinical studies [42]. The psychometric properties of this questionnaire have been examined and verified in terms of reliability, validity, and sensitivity over the years [43]. HAMD has multiple subset scales (Evans-6, MP-6, Toronto-7, Gibbons-8) and various full versions (HAMD-17, HAMD-21, HAMD-24) [44]. In a study, an internal reliability coefficient of 0.83 was obtained for − 17D-HAM and 0.88 for − 24D-HAM [45].

The first standard version of this questionnaire was presented as 17-item HDRS. The scale includes 17 variables that are measured in five-point (0–4) (depressed mood, guilt, suicide, difficulties at work and loss of interests, retardation, anxiety(psychic), anxiety(somatic), hypochondriasis) or three-point (0–2) (insomnia-initial, middle, delayed, agitation, somatic symptoms(gastrointestinal), somatic symptoms(general), genital symptoms, loss of insight, loss of weight) scales. The three-point scale is used in situations where it is difficult to quantify the variable [46]. The severity of depression is considered moderate to high with a total score of 17 and above [47].

In this study, the standard 24-item HDRS was used to evaluate the severity of depression and patients with total scores of 22 and above were included in the study as moderate to severe cases [48]. In this version, other items including Hypersomnia, Social Withdrawal, and Fatigability with a five-point (0–4), Appetite Increase, Increased Eating and Carbohydrate Craving with a four-point (0–3), and Weight Gain with a three-point (0–2) have been added to the previous items.

The Iranian version of this questionnaire has been validated in several studies using different clinical populations and changes in the severity/score of depression following psychological/psychopharmacological interventions [49–53].

#### Side effects checklist

During the clinical trial, we utilized open-ended questions followed by a questionnaire that we designed to evaluate the presence and severity of side effects. Our questionnaire was based on similar ones used for other medications [54–56] and considered the pharmacopeia of empagliflozin [57–59]. We evaluated various items, including:

Headache، Dizzy ، Fainting، Weakness، Nose bleeds، Unusual bleeding، Bruising، Fatigue، Pain، Chills، Nause، Vomiting ، Taste changes ، Mouth sore، Decreased appetite، Stomach cramps، Diarrhea، Constipation، Painful urination، Leaking urine، Frequency of urination، Burning while urinating، Foul smelling urine، Blood in urine (pink /red)، Fever، Hair loss، Change in skin color، Skin dryness، Rash and Itching.

### Procedures

#### Outcome

The primary aim of this study was to investigate the effect of empagliflozin compared to placebo on reducing the severity of depressive symptoms in patients with moderate to severe severity of MDD. Therefore, patients referred to the psychiatric outpatient clinic of the Imam Ali academic Hospital, diagnosed with MDD were evaluated to enter the study. Patients who met the inclusion criteria and did not meet the exclusion criteria were considered for entering the study after the initial evaluation and completion of taking clinical and psychiatric history and providing explanations related to the research project. They entered the study after providing written informed consent. This trial was conducted on patients who were not under related medication or had not taken medication for at least the last two months.

At the beginning of the study and then in the 4th and 8th weeks, the patients were evaluated for the severity of depression based on the HDRS. They were also evaluated using the side-effect checklist in terms of the side effects of taking medication related to the research project.

#### Intervention

After screening and final selection of study participants, patients were randomly divided into two groups receiving placebo or empagliflozin combined with citalopram based on permuted block randomization method. Citalopram was used at first with a dose of 20 mg daily for two weeks and then by increasing the dose to 40 mg daily. Empagliflozin tablets with a dose of 10 mg daily were prescribed in the morning. Placebo tablets with the same condition as empagliflozin tablets in terms of shape, size, and color were given to the other group for daily consumption in the morning. Patients and psychiatrists did not know about the placement of the participants in each of the two groups. Also, they did not know the contents of the pills. All participants in the study were subjected to basic clinical examination and assessment of vital signs (body temperature, pulse rate, respiration rate, blood pressure) before starting the study. The final dose of prescription medication in both groups was the same at the beginning and end of the study.

### Randomization, allocation concealment, and blinding

Participants were randomized using a computer random number generator. In this way, based on a random and unpredictable procedure, the participants were divided into two groups receiving empagliflozin and placebo along with standard treatment. A person who was not a member of the research team and was unaware of the nature of the pills, placed the pills in two groups based on the number of participants, inside separate envelopes unique to each participant. The tablets had the same shape, color and size. Using a random number table, numbers were typed on separate cards according to the number of participants and each was placed in an envelope. Sealed envelopes were prepared and given to the participants after they entered the study. Physicians and patients did not know the nature of the pills. In this way, separate people were randomizing, allocating, and providing pills to patients, none of whom were members of the research study team.

### Data analysis

#### Power analysis

Considering beta less than 20%, and alpha of 5%, the required sample size for the study was estimated to be at least 50 patients. Assuming 20% attrition during the study, the sample size was estimated to be at least 60 patients, and finally, to increase the power of the study, 90 patients (45 patients in each group) were considered as the final sample size.

### Statistical analysis

The quantitative data of this study are shown as mean (standard deviation), and qualitative data are reported as numbers (percentage). At first, the Kolmogorov-Smirnov and Shapiro-Wilk tests were used to check the normal distribution of quantitative data. The data were considered to have a normal distribution if there was a *P* value < 0.05. Due to the normal distribution of data related to Hamilton’s questionnaire in both case and control groups, the two-way repeated-measures ANOVA test (Greenhouse–Geisser correction) was used to analyze the relevant data. Levene’s Test was used to check the Equality of Variance of the age variable and the Chi-Square Test was used to evaluate the equality of qualitative variables between two groups. The analyzes were performed using IBM SPSS Statistics 25.

## Results

**Fig. 1:**
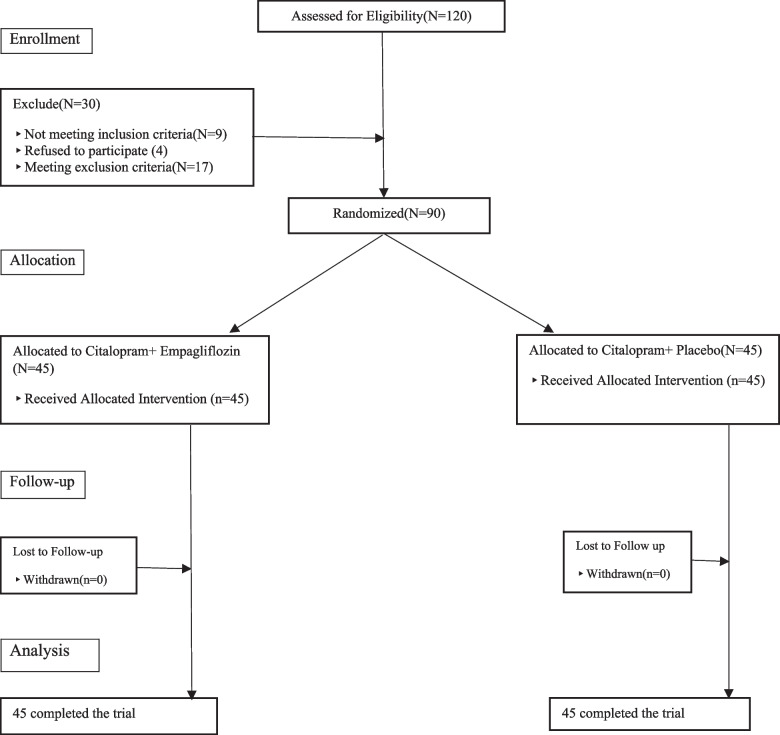
Flow diagram of the clinical trial of the efficacy of empagliflozin on major depressive disorder

120 patients diagnosed with MDD were initially evaluated to enter the study, of which 90 met the inclusion criteria. These participants did not meet the exclusion criteria and were willing to participate in this study. They were included in the study randomly in two groups of 45 patients. None of the participants from both groups withdrew from the study until the end of it (Fig. 1).

The average age of patients receiving placebo was 35.82(± 7.45) versus 34.58(± 8.29) in the group receiving empagliflozin (*p* value = 0.451). 43(47.8%) of the total participants were female and the rest were male. 23(25.6%) of the total participants in the study had a history of suicide attempts and 43(47.8%) had a history of psychiatric hospitalization. Other demographic information and psychiatric records of the patients are specified in Table 1. No statistically significant difference was seen between the two groups in any of the quantitative or qualitative variables.

**Table 1:** Background and demographic characteristics of patients

			Number (percentage) / average (standard deviation)			Chi^2^ /t test	*P* value
			Group A N (%)	Group B N (%)	Total		
Gender	Female		23(53.5)	20(46.5)	43(100.0)	0.401	0.527
	Male		22(46.8)	25(53.2)	47(100.0)		
Job	Housewife		12(41.4)	17(58.6)	29(100.0)	6.387	0.270
	Tradesman		6 (60)	4 (40)	10(100.0)		
	Employee		3(42.9	4(57.1)	7(100.0)		
	Worker		7(36.8)	12(63.2)	19(100.0)		
	Unemployed		16(66.7)	8(33.3)	24(100.0)		
	Retired		1(100.0)	0(0.0)	1(100.0)		
Education	Under-Diploma		19(52.77)	17(47.22)	(100.0)36	1.035	0.793
	High school Diploma		17(44.73)	21(55.26)	38(100.0)		
	Academic Degree		9(56.25)	7(43.75)	16(100.0)		
Marital	Single		14(50.0)	14(50.0)	28(100.0)	2.675	0.445
	Married		21(44.7)	26(57.3)	47(100.0)		
	Widow		6(75.0)	2(25.0)	8(100.0)		
	Divorce		4(57.1)	3(42.9)	7(100)		
Smoking	Positive		24(57.1)	18(42.9)	42(100.0)	1.607	0.205
	Negative		21(43.8)	27(56.3)	48(100.0)		
History of Substance Use		Positive	13(54.2)	11(45.8)	24(100.0)	0227	0.634
		Negative	32(48.5)	34(51.5)	66(100.0)		
Family History of Depression		Positive	21(55.3)	17(44.7)	38(100.0)	0.729	0.393
		Negative	24(46.2)	28(53.8)	52(100.0)		
History of Suicide		Positive	12(52.17)	11(47.82)	23(100.0)	0.058	0.809
		Negative	33(49.25)	34(50.74)	67(100.0)		
History of Psychiatric Admission		Positive	21(48.84)	22(51.16)	43(100.0)	0.045	0.833
		Negative	24(51.06)	23(48.94)	47(100.0)		
Age (years)			34.58(± 8.29)	35.82(± 7.45)	35.20(± 7.87)	(F = 0.574)	0.451

*Group A: Empagliflozin + Citalopram, Group B: Placebo + Citalopram

**Fig. 2:**
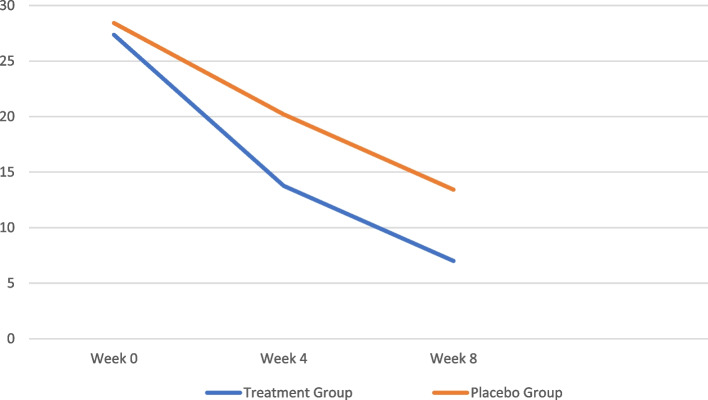
Results of two-factor repeated measure ANOVA for comparison of Hamilton depression rating scale scores (mean ± SEM) over time between the empagliflozin (Treatment) Group and placebo group

Hamilton depression rating scale scores were equal to 28.42(± 3.83), 20.20(± 3.82), and 13.42(± 3.42) in the group that received placebo during weeks 0,4, and 8, respectively. These scores were 27.36(± 3.77), 13.76(± 1.40), and 7.00(± 1.13), respectively, for the group that received empagliflozin (Fig. 2).

Comparative evaluation of HDRS scores among two groups using repeated-measures ANOVA shows a significant difference in scores over time (*p* value = 0.000). Using Greenhouse–Geisser correction, the effect was also significant for time (F (1.653,8664.484) = 976.139, *p* value = 0.000) and time–treatment interaction (F (1.653, 261.394) = 29.449, *p* value = 0.000). In this way, while during the clinical trial, we saw the downward trend of HDRS scores in both arms, the average of these scores in empagliflozin recipients was lower than the placebo group and this was also statistically significant.

Two patients from the group receiving empagliflozin (identification of the group, after the end of the study) complained of urinary symptoms in the form of increased frequency of urination compared to the past without any other urinary symptoms, which was mild and gradually improved without the need for separate diagnostic or therapeutic measures or discontinuation of the medication. No other side effects were reported during the study.

## Discussion

Our study focused on exploring the potential impact of empagliflozin as an additional treatment for patients with moderate to severe Major Depressive Disorder (MDD). The findings we obtained demonstrated the effectiveness of this approach. This outcome can be attributed to the various properties of empagliflozin as an SGLT2 inhibitor, particularly its ability to inhibit the oxidative-inflammatory-apoptosis pathway, and other hormonal effects such as a decrease in serum ACTH levels. Additionally, empagliflozin has neurotransmitter effects such as reducing glutamate concentration [60]. To the best of our knowledge, this study is the first clinical trial that investigates the effect of add-on treatment of empagliflozin with its complex set of neuroreceptor, neuroinflammatory and neurohormonal effects on clinical depression symptoms.

Refardt et al. conducted a study on empagliflozin and its sodium-glucose cotransporter feature. They investigated its role in treating Chronic Syndrome of Inappropriate Antidiuresis. The study showed that after 4 weeks of treatment, the plasma sodium level increased in the group receiving empagliflozin compared to the control group. The trial report indicated that empagliflozin was well-tolerated and didn’t cause any adverse events. The most significant finding of the study was the improved neurocognitive performance in the empagliflozin group as evaluated by the Montreal Cognitive Assessment [MoCA] before and after the 4-week intervention [61].

In some studies, including one on hemodialysis patients, it was found that lower serum sodium levels were significantly associated with depressive symptoms and cognitive deficits in these patients [62]. Additionally, Fujisawa et al. highlighted the link between mild hyponatremia (often without associated clinical symptoms) and depressive mood in a group of elderly people referred to the Memory Disorder Outpatient Center [63]. On the other hand, there have been several reports of hyponatremia related to the use of SSRIs medication [64]. According to a study by Mannheimer et al. on the time course of SSRI-induced hyponatremia, there was a significant increase in the risk of hyponatremia during the first four weeks of SSRI use. This risk then gradually decreased in severity, but even after 13 weeks of first use, the risk remained lower than the control group [65].

It appears that one of the reasons why empagliflozin is beneficial in improving depressive symptoms could be due to its potential to increase serum sodium levels, which could then have a positive effect on the mood and cognitive function of patients. Although we didn’t measure sodium serum levels before and after the intervention during the study, another factor that could have contributed to the improvement of depression in our study compared to the control group is the possible correction of hyponatremia caused by citalopram use. This is particularly important because the risk of hyponatremia tends to increase during the first few weeks of treatment with SSRIs. However, the clinical effects of SSRIs usually take a few weeks to manifest, according to various studies [66].

It has been observed that SSRIs are most effective after a few weeks of continuous usage. However, one of the possible complications that may arise within the first few weeks of starting this medication is hyponatremia. The prevalence of hyponatremia following the use of SSRIs varies depending on study designs, populations, and cutoff values, ranging from 0.06 to 40% in the general population and over 10% among the elderly [67, 68].

It appears that using empagliflozin alongside SSRIs as a starting treatment may have significant clinical benefits, especially in regards to improving depression symptoms. This could be one of the particular reasons why our patients showed more significant progress in comparison to the control group. Furthermore, given that MDD is known to peak in middle age [69] and since our study participants’ average age was 35.20 (± 7.87), the risk of hyponatremia associated with the use of SSRIs in MDD patients is likely higher than in other psychiatric disorders. It is important to note that individuals with Major Depressive Disorder (MDD) often experience a decrease in appetite, resulting in weight loss. This symptom is included in the DSM-5 criteria for diagnosing MDD [70]. Additionally, this group of patients may also have insufficient sodium intake [71].

There are studies that suggest hyponatremia has an effect on the release of glutamate from brain cells into the extracellular space, which in turn contributes to the occurrence of depression symptoms. Anti-glutamate agents are mentioned as a way to reduce these symptoms [63]. There is evidence to suggest that the dysfunction of neuroreceptor systems, specifically glutamatergic neurotransmission via N-methyl-d-aspartate (NMDA) receptors, plays a role in the pathophysiology of depression and suicide. As a result, NMDA receptor antagonists and a group of metabotropic glutamate receptor (mGluR1 and mGluR5) antagonists are being considered as new treatment options for MDD [72–74].

Meanwhile, BDNF plays a significant role in regulating activity-dependent synaptic plasticity [75]. There is a two-way relationship between depression and BDNF expression in the hippocampus and prefrontal cortex. Depression is associated with a decrease in BDNF expression, while antidepressant treatment regulates BDNF signaling and enhances its release [76, 77]. At the neuroreceptor level, BDNF’s specific effect is on the growth and development of glutamatergic and GABA synapses, which affects serotonergic and dopaminergic neurotransmission by modulating neural differentiation [78].

BDNF is a crucial neurotrophic factor that not only affects neuroplasticity but also plays a significant role in the growth and proliferation of neurons and synaptic neurotransmission. According to the neurotrophic theory, a decrease in the level of BDNF in the brain can cause depression [79]. Thus, treatments that increase the level of BDNF can help reduce depressive behavior. Additionally, abnormalities in the serum level of neurotrophins can lead to mood disorders, especially MDD, by causing neuronal atrophy and reducing neurogenesis [2]. Empagliflozin is known for increasing the serum level of BDNF [80], which has been an effective factor in improving the psychiatric condition of patients with MDD. In our study, the group receiving empagliflozin showed a significant improvement in their psychiatric condition compared to the placebo group.

Empagliflozin is a medication that has been found to reduce glutamate concentration. This effect is likely due to its ability to increase serum sodium levels and correct underlying hyponatremia [60]. Glutamate is an excitatory neurotransmitter that interacts with the BDNF system, which is involved in neural plasticity. The two systems have multiple and bidirectional connections, and they mutually regulate each other. Both systems are related to the pathophysiology of depression. When the connections between them are disrupted, it can lead to adverse changes in neural plasticity and ultimately the development of clinical depression [81].

There is growing evidence that suggests a connection between the patterns of gut microbiota and depression. This link is believed to be mediated by the brain-gut microbiome axis [82]. Several studies have found significant differences in microbial taxa between patients with MDD and those in the control group. However, the information available on the microbial diversity, relative abundance, or direction of differences in taxa associated with MDD is limited [83].

Some studies have found that patients with MDD have a higher frequency of pro-inflammatory bacteria like Enterobacteriaceae and Desulfovibrio, and a lower frequency of short-chain fatty acid-producing bacteria like Facalibacterium, compared to control groups [84]. In an animal model, fecal microbiota from patients with MDD was transferred to mice, resulting in depressive-like behavior, decreased levels of hippocampal neurotransmitters, and increased levels of Adrenocorticotropic hormone (ACTH), Corticotropin-releasing hormone (CRH), and serum pro-inflammatory cytokines [85].

Based on the available evidence, one of the significant features of empagliflozin’s add-on treatment is its simultaneous effect on various pathological mechanisms. This includes its impact on BDNF levels, the gut microbiota axis, and its modulatory role on the glutamatergic neuroreceptor system. Our study suggests that this add-on treatment may provide clinical benefits to patients with MDD.

Based on the inflammatory theory of depression, it has been observed that individuals with depression symptoms have higher levels of inflammation, particularly chronic inflammation, when compared to healthy individuals [86]. As a result, the use of anti-inflammatory agents has been reported to have anti-depressant effects. Additionally, numerous reports suggest that using these agents to alleviate symptoms of depression is reasonably safe [17].

In certain cases, when there is an underlying inflammation in the body (indicated by higher serum levels of pro-inflammatory compounds such as CRP/IL-6), patients with MDD may show weaker responses to serotonergic factors. However, they may respond better to antidepressants when these are combined with noradrenergic, dopaminergic, or glutamatergic modulatory agents or anti-inflammatory agents [87]. According to research, the better response of MDD patients to the add-on treatment with empagliflozin in our study could be due to its anti-inflammatory capabilities and modulating effects on the glutamatergic system.

Different results have been reported in human and animal studies regarding the improvement of cognitive status through the use of empagliflozin. Mone et al. reported that in a clinical study of frail older adults with type 2 diabetes and heart failure with preserved ejection fraction, empagliflozin not only improved physical impairment but also cognitive defects. Furthermore, several studies have suggested that empagliflozin may also play a role in reducing brain complications associated with Alzheimer’s disease and type 2 diabetes. Improving endothelial function and reducing mitochondrial oxidative stress have been cited as other possible factors contributing to the improvement of cognitive status in individuals treated with empagliflozin [88–91].

Cognitive function impairment and information processing deficit are common features of depression and brain disorders, which are mediated by GABA defects [92]. In contrast, agents that inhibit SGLT2 and activate GABA are shown to increase BDNF production [93]. Therefore, empagliflozin can potentially improve depression symptoms by directly influencing the BDNF system, and indirectly affecting cognitive functions through GABA activation. This association is also observed in other classes of antidepressants, such as Selective Serotonin Reuptake Enhancers (SSREs)-tianeptine, the Serotonin and Norepinephrine Reuptake Inhibitors (SNRIs)-duloxetine, vortioxetine, as well as other antidepressants like bupropion and moclobemide. The association between cognitive dysfunction and MDD is so strong that in some studies, they are referred to as a state and a trait marker of depression [94].

Looking at it one way, there are certain functional similarities between empagliflozin and certain types of antidepressants. However, most antidepressant treatments are not intended to target cognition specifically [95]. Additionally, there is contradictory evidence regarding their effectiveness and potential negative impact on the cognitive wellbeing of patients with MDD [96].

In a case report from Japan, a 55-year-old female patient with type 2 diabetes from 3 years before the recent admission and with a 5-year history of a depressive state, suffered from exacerbation of underlying diabetes symptoms. After the required evaluations, in addition to the previous antidiabetic treatments (including metformin and vildagliptin), one of the SGLT2 inhibitor drugs (ipragliflozin l-proline) was added to the treatment regimen. This patient was being treated with duloxetine hydrochloride and mirtazapine due to depression and occasional suicidal thoughts. In the next follow-up, although a quantitative evaluation of the severity of the patient’s depression was not performed, some depressive symptoms such as suicidal thoughts gradually disappeared after starting the administration of ipragliflozin, and also, proper control of the patient’s blood sugar status was achieved [97].

The results of this case report are in line with our study’s findings on the effectiveness of empagliflozin in reducing symptoms of depression in patients with MDD. The patients involved in the 8-week trial did not have diabetes as a comorbidity and were able to tolerate the medication without experiencing any serious side effects. This outcome suggests that empagliflozin could be beneficial for treating depressive disorders in non-diabetic patients.

In terms of the positive effect of empagliflozin on depressive symptoms, the results of our study have similarities with the results of the clinical trial conducted by ŞAHİN et al., who reported an improvement in the quality of life in patients with type 2 diabetes treated for 3 months with empagliflozin/dapagliflozin compared to the control group. Such a positive effect has been seen in an improvement in mental health, vitality, and emotional role limitation in the treated group compared to the control group [40]. Although in this study, the effect of empagliflozin/dapagliflozin on depression was not evaluated and no positive effect of this treatment on anxiety was obtained.

In a recent study, it was found that administering 25 mg of empagliflozin daily for a period of 3 months, along with triple drug therapy (metformin, teneligliptin, and glimepiride), resulted in significant improvement in the quality of life of patients suffering from type 2 diabetes mellitus with hypertension. The study reported improvements in emotional/mental health and physical health [98]. Not just diabetic patients, but non-diabetic patients with heart failure with reduced ejection fraction (HFrEF) also reported improvements in their quality of life after being administered empagliflozin [99].

### Limitations

There are several underlying mechanisms for the possible effect of empagliflozin on reducing depression symptoms. In this study, we evaluated the severity of depression symptoms based on the HDRS, and laboratory evaluations, including the measurement of serum levels of sodium, inflammatory factors, and some hormones, were not performed in this study. Such evaluations can be part of the design of future studies to explain more precisely the mechanisms involved in the effect of empagliflozin on depression symptoms.

Also, investigating possible changes in the density and diversity of gut microbiota following 8-week treatment with empagliflozin can be considered in future studies in order to evaluate the relationship between these changes and improvement in depression symptoms. Paying attention to cognitive changes along with changes in the severity of depression symptoms due to the possible two-way relationship of these changes in patients with MDD, can be one of the topics of interest in future studies in this field and was not evaluated in this study.

In this study, we compared the severity of depression between two groups. To compare the effectiveness of treatment methods in addition to the analysis of reducing the severity of depression, it is also possible to compare the rate of therapeutic response and remission at the end of the observation. Such a study design can be done under the next studies in this regard.

The existing evidence of the correlation between glucose metabolism and mechanisms of MDD is insufficient. Therefore, it cannot be assumed that the significant decrease in scale scores in this study is entirely associated with the intervention of empagliflozin. The eliminating knowledge gaps in this field in the future and conducting similar studies with larger sample sizes may be a way forward in this field.

In our study, we only considered moderate to severe side effects that were rated as 4 or higher on a scale of 0 to 10. However, if the symptoms persisted for more than a week, we also recorded cases with a symptom intensity of less than 4. We closely monitored all cases of mild to severe side effects and evaluated them on a daily basis in terms of their severity, recovery process, and any need for therapeutic intervention.

## Conclusions

In this clinical trial, empagliflozin was used as an add-on treatment to citalopram among patients with MDD. It works as a highly selective and potent inhibitor of sodium-glucose co-transporter 2 (SGLT2). The study found that this medication was associated with psychiatric usefulness and a reduction in the severity of depression symptoms compared to the control group. Considering the multitude of possible mechanisms involved in the formation of this effect along with the metabolic and vascular benefits of this medication, it seems that evaluating the effect of empagliflozin as an adjuvant treatment on MDD among different clinical groups with a larger sample size and in multicenter conditions with longer follow-ups may be accompanied by more accurate clinical judgment in this field.

## Data Availability

The datasets used and/or analyzed during the current study are available from the corresponding author (Rahim Badrfam) on reasonable request.

## References

[1] Gutiérrez-Rojas L, Porras-Segovia A, Dunne H, Andrade-González N, Cervilla JA (2020). Prevalence and correlates of major depressive disorder: a systematic review. Brazilian J Psychiatry.

[2] Mosiołek A, Mosiołek J, Jakima S, Pięta A, Szulc A (2021). Effects of antidepressant treatment on neurotrophic factors (BDNF and IGF-1) in patients with major depressive disorder (MDD). J Clin Med.

[3] Ruhe HG, Mocking RJ, Figueroa CA, Seeverens PW, Ikani N, Tyborowska A (2019). Emotional biases and recurrence in major depressive disorder. Results of 2.5 years follow-up of drug-free cohort vulnerable for recurrence. Front Psychiatry.

[4] Herrman H, Kieling C, McGorry P, Horton R, Sargent J, Patel V (2019). Reducing the global burden of depression: a Lancet–World Psychiatric Association Commission. Lancet.

[5] Harris MG, Kazdin AE, Chiu WT, Sampson NA, Aguilar-Gaxiola S, Al-Hamzawi A (2020). Findings from world mental health surveys of the perceived helpfulness of treatment for patients with major depressive disorder. JAMA Psychiat.

[6] Ghaffari Darab M, Hedayati A, Khorasani E, Bayati M, Keshavarz K (2020). Selective serotonin reuptake inhibitors in major depression disorder treatment: an umbrella review on systematic reviews. Int J Psychiatry Clin Pract.

[7] Hutchison SM, Mâsse LC, Pawluski JL, Oberlander TF (2021). Perinatal selective serotonin reuptake inhibitor (SSRI) and other antidepressant exposure effects on anxiety and depressive behaviors in offspring: a review of findings in humans and rodent models. Reprod Toxicol.

[8] Zhao B, Li Z, Wang Y, Ma X, Wang X, Wang X (2019). Manual or electroacupuncture as an add-on therapy to SSRIs for depression: a randomized controlled trial. J Psychiatr Res.

[9] Braun C, Adams A, Rink L, Bschor T, Kuhr K, Baethge C (2020). In search of a dose–response relationship in SSRIs—a systematic review, meta-analysis, and network meta‐analysis. Acta Psychiatr Scand.

[10] Furukawa TA, Cipriani A, Cowen PJ, Leucht S, Egger M, Salanti G (2019). Optimal dose of selective serotonin reuptake inhibitors, venlafaxine, and mirtazapine in major depression: a systematic review and dose-response meta-analysis. Lancet Psychiatry.

[11] Duman RS, Deyama S, Fogaça MV (2021). Role of BDNF in the pathophysiology and treatment of depression: activity-dependent effects distinguish rapid‐acting antidepressants. Eur J Neurosci.

[12] Siwek M, Sowa-Kućma M, Dudek D, Styczeń K, Szewczyk B, Kotarska K (2013). Oxidative stress markers in affective disorders. Pharmacol Rep.

[13] Miller AH, Raison CL (2016). The role of inflammation in depression: from evolutionary imperative to modern treatment target. Nat Rev Immunol.

[14] Krupa AJ, Dudek D, Siwek M (2024). Consolidating evidence on the role of insulin resistance in major depressive disorder. Curr Opin Psychiatry.

[15] Fernandes BS, Salagre E, Enduru N, Grande I, Vieta E, Zhao Z (2022). Insulin resistance in depression: a large meta-analysis of metabolic parameters and variation. Neurosci Biobehav Rev.

[16] Dome P, Tombor L, Lazary J, Gonda X, Rihmer Z (2019). Natural health products, dietary minerals and over-the-counter medications as add-on therapies to antidepressants in the treatment of major depressive disorder: a review. Brain Res Bull.

[17] Bai S, Guo W, Feng Y, Deng H, Li G, Nie H (2020). Efficacy and safety of anti-inflammatory agents for the treatment of major depressive disorder: a systematic review and meta-analysis of randomised controlled trials. J Neurol Neurosurg Psychiatry.

[18] Abdallah MS, Mosalam EM, Zidan A-AA, Elattar KS, Zaki SA, Ramadan AN (2020). The antidiabetic metformin as an adjunct to antidepressants in patients with major depressive disorder: a proof-of-concept, randomized, double-blind, placebo-controlled trial. Neurotherapeutics.

[19] Sepanjnia K, Modabbernia A, Ashrafi M, Modabbernia M-J, Akhondzadeh S (2012). Pioglitazone adjunctive therapy for moderate-to-severe major depressive disorder: randomized double-blind placebo-controlled trial. Neuropsychopharmacology.

[20] Chung YJ, Park KC, Tokar S, Eykyn TR, Fuller W, Pavlovic D (2021). Off-target effects of sodium-glucose co-transporter 2 blockers: empagliflozin does not inhibit Na+/H + exchanger-1 or lower [Na+] i in the heart. Cardiovascular Res.

[21] Tan Y, Yu K, Liang L, Liu Y, Song F, Ge Q (2021). Sodium–glucose co-transporter 2 inhibition with empagliflozin improves cardiac function after cardiac arrest in rats by enhancing mitochondrial energy metabolism. Front Pharmacol.

[22] Jamalizadeh M, Hasanzad M, Sarhangi N, Sharifi F, Nasli-Esfahani E, Larijani B (2021). Pilot study in pharmacogenomic management of empagliflozin in type 2 diabetes mellitus patients. J Diabetes Metab Disorders.

[23] Arun S, Praveen D, Chowdary RP, Aanandhi VM (2022). A Comprehensive Review on Sodium glucose co-transporter-2 inhibitors-Empagliflozin. Res J Pharm Technol.

[24] Santos-Gallego CG, Requena-Ibanez JA, San Antonio R, Ishikawa K, Watanabe S, Picatoste B (2019). Empagliflozin ameliorates adverse left ventricular remodeling in nondiabetic heart failure by enhancing myocardial energetics. J Am Coll Cardiol.

[25] Fitchett D, Inzucchi SE, Cannon CP, McGuire DK, Scirica BM, Johansen OE (2019). Empagliflozin reduced mortality and hospitalization for heart failure across the spectrum of cardiovascular risk in the EMPA-REG OUTCOME trial. Circulation.

[26] Abdel-Latif RG, Rifaai RA, Amin EF (2020). Empagliflozin alleviates neuronal apoptosis induced by cerebral ischemia/reperfusion injury through HIF-1α/VEGF signaling pathway. Arch Pharm Res.

[27] Amin EF, Rifaai RA, Abdel-latif RG (2020). Empagliflozin attenuates transient cerebral ischemia/reperfusion injury in hyperglycemic rats via repressing oxidative–inflammatory–apoptotic pathway. Fundam Clin Pharmacol.

[28] Li Y, Wang M-L, Zhang B, Fan X-X, Tang Q, Yu X (2022). Antidepressant-like effect and mechanism of ginsenoside rd on rodent models of depression. Drug Des Dev Ther.

[29] Fan X-X, Sun W-Y, Li Y, Tang Q, Li L-N, Yu X (2022). Honokiol improves depression-like behaviors in rats by HIF-1α-VEGF signaling pathway activation. Front Pharmacol.

[30] Kobayashi K, Toyoda M, Hatori N (2019). Clinical comparison of tofogliflozin and empagliflozin based on an analysis of 24-h accumulated urine in Japanese patients with type 2 diabetes mellitus. Obes Med.

[31] Choi KW, Na EJ, Fava M, Mischoulon D, Cho H, Jeon HJ (2018). Increased adrenocorticotropic hormone (ACTH) levels predict severity of depression after six months of follow-up in outpatients with major depressive disorder. Psychiatry Res.

[32] Parker KJ, Schatzberg AF, Lyons DM (2003). Neuroendocrine aspects of hypercortisolism in major depression. Horm Behav.

[33] Higashikawa T, Ito T, Mizuno T, Ishigami K, Kuroki K, Maekawa N (2021). Effects of tofogliflozin on adrenocorticotropic hormone, renin and aldosterone, and cortisol levels in elderly patients with diabetes mellitus: a retrospective study of a patient cohort. Medicine.

[34] Beurel E, Toups M, Nemeroff CB (2020). The bidirectional relationship of depression and inflammation: double trouble. Neuron.

[35] Heimke M, Lenz F, Rickert U, Lucius R, Cossais F (2022). Anti-inflammatory properties of the SGLT2 inhibitor empagliflozin in activated primary Microglia. Cells.

[36] Abdelhamid AM, Elsheakh AR, Abdelaziz RR, Suddek GM (2020). Empagliflozin ameliorates ethanol-induced liver injury by modulating NF-κB/Nrf-2/PPAR-γ interplay in mice. Life Sci.

[37] Wang X-q, Tang Y-h, Zeng G-r, Wu L-f, Zhou Y-j, Cheng Z-n (2021). Carnosic acid alleviates depression-like behaviors on chronic mild stressed mice via PPAR-γ-dependent regulation of ADPN/FGF9 pathway. Psychopharmacology.

[38] Cigliano L, Spagnuolo MS, Boscaino F, Ferrandino I, Monaco A, Capriello T (2019). Dietary supplementation with fish oil or conjugated linoleic acid relieves depression markers in mice by modulation of the Nrf2 pathway. Mol Nutr Food Res.

[39] Subba R, Ahmad MH, Ghosh B, Mondal AC (2022). Targeting NRF2 in type 2 diabetes mellitus and depression: efficacy of natural and synthetic compounds. Eur J Pharmacol.

[40] Şahin S, Haliloğlu Ö, Korkmaz ÖP, Durcan E, Şahİn HR, Yumuk VD (2021). Does treatment with sodium-glucose co-transporter-2 inhibitors have an effect on sleepquality, quality of life, and anxiety levels in people with type 2 diabetes mellitus?. Turk J Med Sci.

[41] Klarskov CK, Holm Schultz H, Persson F, Møller Christensen T, Almdal TP, Snorgaard O (2020). Study rationale and design of the EANITIATE study (EmpAgliflozin compared to NPH insulin for sTeroId diAbeTEs)-a randomized, controlled, multicenter trial of safety and efficacy of treatment with empagliflozin compared with NPH-insulin in patients with newly onset diabetes following initiation of glucocorticoid treatment. BMC Endocr Disorders.

[42] Williams JB (1988). A structured interview guide for the Hamilton Depression Rating Scale. Arch Gen Psychiatry.

[43] Williams JB (2001). Standardizing the Hamilton Depression Rating Scale: past, present, and future. Eur Arch Psychiatry Clin NeuroSci.

[44] Fenton C, McLoughlin DM (2021). Usefulness of Hamilton rating scale for depression subset scales and full versions for electroconvulsive therapy. PLoS ONE.

[45] Akdemir A, Örsel DS, Dağ İ, Türkçapar MH (1996). Hamilton depresyon derecelendirme ölçeği (HDDÖ)’nin geçerliliği-güvenirliliği ve klinikte kullanımı. Psikiyatri Psikoloji Psikofarmakoloji Dergisi.

[46] Hamilton M (1960). A rating scale for depression. J Neurol Neurosurg Psychiatry.

[47] Zimmerman M, Martinez JH, Young D, Chelminski I, Dalrymple K (2013). Severity classification on the Hamilton depression rating scale. J Affect Disord.

[48] Roose SP, Sackeim HA, Krishnan KRR, Pollock BG, Alexopoulos G, Lavretsky H (2004). Antidepressant pharmacotherapy in the treatment of depression in the very old: a randomized, placebo-controlled trial. Am J Psychiatry.

[49] Sadeghi A, Ghorayshi F, Baghshahi H, Akbari H, Memarzadeh MR, Taghizadeh M (2023). The antidepressant effect of combined extracts of Hypericum perforatum and Echium amoenum supplementation in patients with depression symptoms: a randomized clinical trial. Avicenna J Phytomed.

[50] Moradveisi L, Huibers MJ, Renner F, Arasteh M, Arntz A (2013). The influence of comorbid personality disorder on the effects of behavioural activation vs. antidepressant medication for major depressive disorder: results from a randomized trial in Iran. Behav Res Ther.

[51] Mirabdolhagh Hazaveh M, Dormohammadi Toosi T, Nasiri Toosi M, Tavakoli A, Shahbazi F (2015). Prevalence and severity of depression in chronic viral hepatitis in Iran. Gastroenterol Rep.

[52] Baghestani S, Zare S, Seddigh SH (2015). Severity of depression and anxiety in patients with Alopecia Areata in Bandar Abbas Iran. Dermatology Rep.

[53] Dehesh T, Dehesh P, Shojaei S (2020). Prevalence and associated factors of anxiety and depression among patients with type 2 diabetes in Kerman, Southern Iran. Diabetes Metab Syndr Obesity.

[54] Ashoorian D, Davidson R, Rock D, Dragovic M, Clifford R (2015). A clinical communication tool for the assessment of psychotropic medication side effects. Psychiatry Res.

[55] Short B, Fong J, Galvez V, Shelker W, Loo CK (2018). Side-effects associated with ketamine use in depression: a systematic review. Lancet Psychiatry.

[56] Hetrick SE, Dellosa MK, Simmons MB, Phillips L (2015). Development and pilot testing of an online monitoring tool of depression symptoms and side effects for young people being treated for depression. Early Interv Psychiat.

[57] Manoel JW, Primieri GB, Bueno LM, Wingert NR, Volpato NM, Garcia CV (2020). The application of quality by design in the development of the liquid chromatography method to determine empagliflozin in the presence of its organic impurities. RSC Adv.

[58] Scheen AJ (2014). Pharmacokinetic and pharmacodynamic profile of empagliflozin, a sodium glucose co-transporter 2 inhibitor. Clin Pharmacokinet.

[59] Heise T, Seman L, Macha S, Jones P, Marquart A, Pinnetti S (2013). Safety, tolerability, pharmacokinetics, and pharmacodynamics of multiple rising doses of empagliflozin in patients with type 2 diabetes mellitus. Diabetes Therapy.

[60] Avgerinos KI, Mullins RJ, Vreones M, Mustapic M, Chen Q, Melvin D (2022). Empagliflozin Induced Ketosis, upregulated IGF-1/Insulin receptors and the Canonical Insulin Signaling Pathway in neurons, and decreased the excitatory neurotransmitter glutamate in the brain of non-diabetics. Cells.

[61] Refardt J, Imber C, Nobbenhuis R, Sailer CO, Haslbauer A, Monnerat S (2022). Treatment effect of the SGLT2 inhibitor empagliflozin on chronic syndrome of inappropriate antidiuresis: results of a randomized, double-blind, placebo-controlled, crossover trial. J Am Soc Nephrol.

[62] Fan SS, Lin LF, Chen VCH, Hsieh CW, Hsiao HP, McIntyre RS (2020). Effects of Lower Past-Year serum sodium and hyponatremia on depression symptoms and cognitive impairments in patients with hemodialysis. Ther Apher Dial.

[63] Fujisawa C, Umegaki H, Sugimoto T, Samizo S, Huang CH, Fujisawa H (2021). Mild hyponatremia is associated with low skeletal muscle mass, physical function impairment, and depressive mood in the elderly. BMC Geriatr.

[64] Kim G-H (2022). Pathophysiology of Drug-Induced Hyponatremia. J Clin Med.

[65] Mannheimer B, Falhammar H, Calissendorff J, Skov J, Lindh JD (2021). Time-dependent association between selective serotonin reuptake inhibitors and hospitalization due to hyponatremia. J Psychopharmacol.

[66] Michely J, Eldar E, Martin IM, Dolan RJ (2020). A mechanistic account of serotonin’s impact on mood. Nat Commun.

[67] Mannesse CK, Jansen PA, Van Marum RJ, Sival RC, Kok RM, Haffmans PJ (2013). Characteristics, prevalence, risk factors, and underlying mechanism of hyponatremia in elderly patients treated with antidepressants: a cross-sectional study. Maturitas.

[68] De Picker L, Van Den Eede F, Dumont G, Moorkens G, Sabbe BG (2014). Antidepressants and the risk of hyponatremia: a class-by-class review of literature. Psychosomatics.

[69] Bogren M, Brådvik L, Holmstrand C, Nöbbelin L, Mattisson C (2018). Gender differences in subtypes of depression by first incidence and age of onset: a follow-up of the Lundby population. Eur Arch Psychiatry Clin NeuroSci.

[70] Tolentino JC, Schmidt SL (2018). DSM-5 criteria and depression severity: implications for clinical practice. Front Psychiatry.

[71] Bryant E (2021). Anorexia: the great taboo. Lancet Psychiatry.

[72] Paul IA, Skolnick P (2003). Glutamate and depression: clinical and preclinical studies. Ann N Y Acad Sci.

[73] Duman RS, Sanacora G, Krystal JH (2019). Altered connectivity in depression: GABA and glutamate neurotransmitter deficits and reversal by novel treatments. Neuron.

[74] Amidfar M, Woelfer M, Reus GZ, Quevedo J, Walter M, Kim Y-K (2019). The role of NMDA receptor in neurobiology and treatment of major depressive disorder: evidence from translational research. Prog Neuropsychopharmacol Biol Psychiatry.

[75] Keifer J (2022). Regulation of AMPAR trafficking in synaptic plasticity by BDNF and the impact of neurodegenerative disease. J Neurosci Res.

[76] Chen C, Dong Y, Liu F, Gao C, Ji C, Dang Y (2020). A study of antidepressant effect and mechanism on intranasal delivery of BDNF-HA2TAT/AAV to rats with post-stroke depression. Neuropsychiatr Dis Treat.

[77] Castrén E, Monteggia LM (2021). Brain-derived neurotrophic factor signaling in depression and antidepressant action. Biol Psychiatry.

[78] Colucci-D’Amato L, Speranza L, Volpicelli F (2020). Neurotrophic factor BDNF, physiological functions and therapeutic potential in depression, neurodegeneration and brain cancer. Int J Mol Sci.

[79] Rana T, Behl T, Sehgal A, Srivastava P, Bungau S (2021). Unfolding the role of BDNF as a biomarker for treatment of Depression. J Mol Neurosci.

[80] Mousa HH, Sharawy MH, Nader MA (2023). Empagliflozin enhances neuroplasticity in rotenone-induced parkinsonism: role of BDNF, CREB and Npas4. Life Sci.

[81] Gulyaeva N (2017). Interplay between brain BDNF and glutamatergic systems: a brief state of the evidence and association with the pathogenesis of depression. Biochem (Moscow).

[82] Barandouzi ZA, Starkweather AR, Henderson WA, Gyamfi A, Cong XS (2020). Altered composition of gut microbiota in depression: a systematic review. Front Psychiatry.

[83] Cheung SG, Goldenthal AR, Uhlemann A-C, Mann JJ, Miller JM, Sublette ME (2019). Systematic review of gut microbiota and major depression. Front Psychiatry.

[84] Simpson CA, Diaz-Arteche C, Eliby D, Schwartz OS, Simmons JG, Cowan CS (2021). The gut microbiota in anxiety and depression–a systematic review. Clin Psychol Rev.

[85] Liu S, Guo R, Liu F, Yuan Q, Yu Y, Ren F (2020). Gut microbiota regulates depression-like behavior in rats through the neuroendocrine-immune-mitochondrial pathway. Neuropsychiatr Dis Treat.

[86] Carniel BP, da Rocha NS (2021). Brain-derived neurotrophic factor (BDNF) and inflammatory markers: perspectives for the management of depression. Prog Neuropsychopharmacol Biol Psychiatry.

[87] Arteaga-Henríquez G, Simon MS, Burger B, Weidinger E, Wijkhuijs A, Arolt V (2019). Low-grade inflammation as a predictor of antidepressant and anti-inflammatory therapy response in MDD patients: a systematic review of the literature in combination with an analysis of experimental data collected in the EU-MOODINFLAME consortium. Front Psychiatry.

[88] Mone P, Lombardi A, Gambardella J, Pansini A, Macina G, Morgante M (2022). Empagliflozin improves cognitive impairment in frail older adults with type 2 diabetes and heart failure with preserved ejection fraction. Diabetes Care.

[89] Hierro-Bujalance C, Infante-Garcia C, Del Marco A, Herrera M, Carranza-Naval MJ, Suarez J (2020). Empagliflozin reduces vascular damage and cognitive impairment in a mixed murine model of Alzheimer’s disease and type 2 diabetes. Alzheimers Res Ther.

[90] Khan T, Khan S, Akhtar M, Ali J, Najmi AK (2021). Empagliflozin nanoparticles attenuates type2 diabetes induced cognitive impairment via oxidative stress and inflammatory pathway in high fructose diet induced hyperglycemic mice. Neurochem Int.

[91] Mone P, Varzideh F, Jankauskas SS, Pansini A, Lombardi A, Frullone S (2022). SGLT2 inhibition via empagliflozin improves endothelial function and reduces mitochondrial oxidative stress: insights from frail hypertensive and diabetic patients. Hypertension.

[92] Prévot T, Sibille E (2021). Altered GABA-mediated information processing and cognitive dysfunctions in depression and other brain disorders. Mol Psychiatry.

[93] Kamel AS, Wahid A, Abdelkader NF, Ibrahim WW (2022). Boosting amygdaloid GABAergic and neurotrophic machinery via dapagliflozin-enhanced LKB1/AMPK signaling in anxious demented rats. Life Sci.

[94] Baune BT, Renger L (2014). Pharmacological and non-pharmacological interventions to improve cognitive dysfunction and functional ability in clinical depression–a systematic review. Psychiatry Res.

[95] Kaser M, Zaman R, Sahakian BJ (2017). Cognition as a treatment target in depression. Psychol Med.

[96] Marazziti D, Mucci F, Tripodi B, Carbone MG, Muscarella A, Falaschi V (2019). Emotional blunting, cognitive impairment, bone fractures, and bleeding as possible side effects of long-term use of SSRIs. Clin Neuropsychiatr.

[97] Shimizu E, Takehisa Y, Bando H, Fujita M, Kusaka Y, Yuu M (2020). Effective SGLT2 inhibitor for patient with type 2 diabetes Mellitus (T2DM) and Depression. Diabetes Res.

[98] Najar IA, Masoodi SR, Mir SA, Bhat MH, Patyar RR, Patyar S (2022). Impact of empagliflozin add-on therapy on quality of life in patients of type 2 diabetes mellitus with hypertension: a prospective study. Indian J Public Health.

[99] Requena-Ibáñez JA, Santos-Gallego CG, Rodriguez-Cordero A, Vargas-Delgado AP, Badimón JJ (2022). Empagliflozin improves quality of life in nondiabetic HFrEF patients. Sub-analysis of the EMPATROPISM trial. Diabetes Metab Syndr.

